# Thioredoxin Inhibitors Attenuate Platelet Function and Thrombus Formation

**DOI:** 10.1371/journal.pone.0163006

**Published:** 2016-10-07

**Authors:** Clive Metcalfe, Anjana Ramasubramoni, Giordano Pula, Matthew T. Harper, Stuart J. Mundell, Carmen H. Coxon

**Affiliations:** 1 Oxford Molecular and Pathology Institute, South Parks Road, Oxford, OX1 3RE, United Kingdom; 2 Department of Pharmacy and Pharmacology, University of Bath, Claverton Down, Bath, BA2 7AY, United Kingdom; 3 Department of Pharmacology, University of Cambridge, Tennis Court Road, Cambridge, CB2 1PD, United Kingdom; 4 Department of Physiology, Pharmacology and Neuroscience, Medical Sciences Building, University of Bristol, University Walk, Bristol, BS8 1TD, United Kingdom; Queen Mary University of London, UNITED KINGDOM

## Abstract

Thioredoxin (Trx) is an oxidoreductase with important physiological function. Imbalances in the NADPH/thioredoxin reductase/thioredoxin system are associated with a number of pathologies, particularly cancer, and a number of clinical trials for thioredoxin and thioredoxin reductase inhibitors have been carried out or are underway. Due to the emerging role and importance of oxidoreductases for haemostasis and the current interest in developing inhibitors for clinical use, we thought it pertinent to assess whether inhibition of the NADPH/thioredoxin reductase/thioredoxin system affects platelet function and thrombosis. We used small molecule inhibitors of Trx (PMX 464 and PX-12) to determine whether Trx activity influences platelet function, as well as an unbiased proteomics approach to identify potential Trx substrates on the surface of platelets that might contribute to platelet reactivity and function. Using LC-MS/MS we found that PMX 464 and PX-12 affected the oxidation state of thiols in a number of cell surface proteins. Key surface receptors for platelet adhesion and activation were affected, including the collagen receptor GPVI and the von Willebrand factor receptor, GPIb. To experimentally validate these findings we assessed platelet function in the presence of PMX 464, PX-12, and rutin (a selective inhibitor of the related protein disulphide isomerase). In agreement with the proteomics data, small molecule inhibitors of thioredoxin selectively inhibited GPVI-mediated platelet activation, and attenuated ristocetin-induced GPIb-vWF-mediated platelet agglutination, thus validating the findings of the proteomics study. These data reveal a novel role for thioredoxin in regulating platelet reactivity via proteins required for early platelet responses at sites of vessel injury (GPVI and GPIb). This work also highlights a potential opportunity for repurposing of PMX 464 and PX-12 as antiplatelet agents.

## Introduction

Oxidation/reduction of disulphide bonds contributes to cell viability and survival. Disruption of this system can have a significant impact on physiology and disease. The NADPH/thioredoxin reductase/thioredoxin system largely oversees cellular reduction/oxidation balance in the cell, with glutathione/glutathione reductase and other enzymes (protein disulphides isomerases (PDIs), peroxireducatses etc) also playing a role. Together, these enzyme systems oversee and regulate oxidation/reduction balance, scavenge reactive oxygen species, contribute to protein folding in the endoplasmic reticulum, and regulate the activity of a number of proteins involved in DNA repair, apoptosis, and transcription[1–6]. In addition to these intracellular roles, these enzyme systems can also regulate extracellular processes via effects on catalytic and allosteric disulphides bonds in their substrates. Both PDI and thioredoxin (Trx) have been shown to influence a myriad of extracellular processes, including HIV infection[7, 8], integrin activation[9, 10], receptor-ligand interactions[7, 11], and thrombus formation[12, 13]. These functions influence a number of pathophysiological processes [14–16], particularly cancer [17–20], where enhanced Trx-1 levels and activity promotes tumor cell growth and survival *in vitro*, while loss of Trx-1 or its activity promotes apoptosis and prevents tumor formation in mice. This correlation between Trx and disease has lead to the development of a number of Trx inhibitors that we have exploited to use as chemical tools to investigate the role of Trx in platelet function.

Initially we found that inhibition of Trx activity affects the profile of reduced thiols at the surface of resting platelets, following which we used a proteomics approach to determine which proteins were affected. We identified key platelet adhesion/activation receptors whose thiol profile was altered by Trx inhibition, and subsequent investigation correlated these changes with changes in protein function and platelet activation via the collagen (GPVI) and von Willebrand (GPIb-IX-V) receptors (thrombin, ADP and thromboxane A2 receptors were not affected). Ristocetin-induced platelet agglutination and CRP-XL-induced platelet activation was impaired by Trx inhibitors, as was thrombus formation on type I collagen in whole blood under flow. This indicates that the NADPH/Trx-R/Trx system influences platelet reactivity to immobilized ligands (vWF and collagen) at sites of injury and demonstrates for the first time that redox modulation of platelet cell surface proteins by thiol isomerases *other than PDIs* is important for platelet function in the early response to injury. As inhibition of GPVI-collagen and GPIb-vWF interactions is known to reduce pathological thrombosis with minimal effect on the physiological response to injury[21], especially with regards to ischemic stroke, there is considerable interest in exploiting these interactions for drug development. Inhibitors of the NADPH/Trx-R/Trx system developed to treat cancer and known to be well tolerated in man may be appropriate for repurposing an antiplatelet drugs.

## Methods

### Materials

Anti-CD42b was purchased from Santa Cruz Biotechnology. Alexa Fluor 647 anti-GPVI antibody (clone HY101) was purchased from BD Pharmingen. Auranofin, PX-12, PMX 464, and U46619 were purchased from Tocris Bioscience (Bristol, U.K.). All other reagents were purchased from Sigma (Poole, U.K.). Collagen-related peptide (GCO-[GPO]_10_GCOG-NH2) was synthesised and cross-linked by Peptide Protein Research Ltd (Cambridge UK).

### Preparation of washed human platelets

Whole blood was taken from healthy volunteers (from which informed, written consent was obtained) and collected into 50 ml syringes containing 5 ml 4% sodium citrate in accordance with procedures approved by the Local Research Ethics Committee (Faculty of Medical and Veterinary Sciences Ethics Committee, United Bristol Healthcare Trust project number D5736). Acid citrate dextrose (ACD; 0.15% (w/v) citric acid, 0.4% (w/v) trisodium citrate dihydrate, 0.2% (w/v) glucose) was added (1/7) to citrated blood and mixed by gentle inversion before platelet-rich plasma (PRP) was isolated by centrifugation at 200 *x*g for 17 minutes at room temperature. PRP was pooled and 0.5 μM PGI_2_ was added before centrifugation at 1000 *x*g for 10 minutes at room temperature. Platelets were resuspended in 1ml Tyrodes-HEPES buffer (134 mM NaCl, 0.34 mM Na_2_HPO_4_, 2.9 mM KCl, 12 mM NaHCO_3_, 20 mM 4-(2-hydroxyethyl)-1-piperazineethanesulfonic acid (HEPES), 5 mM glucose, 1 mM MgCl_2_, pH 7.3, pre-warmed to 30°C and 150 μl ACD. The volume was adjusted to 25ml with Tyrodes-HEPES buffer, followed by addition of 3 ml ACD and 0.5 μM PGI_2_. Cells were centrifuged at 1000 *x*g for 10 minutes at room temperature and the resultant cell pellet was resuspended in pre-warmed Tyrodes buffer to a final cell density of 4x10^8^ cells/ml (aggregations) or 2x10^9^ cells/ml (peptide pull downs) and rested for at least 30 minutes before use. Where appropriate, 1 mM ethylene glycol tetraacetic acid (EGTA), 10 μM indomethacin and 2 U/ml apyrase were added to inhibit platelet aggregation (referred to as non-aggregating conditions).

### Isolation of platelet-rich plasma from whole blood

Platelet-rich plasma was separated from citrated whole blood (without ACD) by centrifugation at 200 *x*g for 20 minutes.

### Light transmission aggregometry

Platelets or PRP (250 μl) were stimulated with agonist at 37°C with continuous stirring (1200 rpm) in an optical aggregometer. For drug studies, platelets or PRP were incubated with drug under non-stirring conditions at 30°C for the desired time prior to the addition of agonist under stirring conditions. Aggregation was monitored using AGRO/LINK8 software (Chrono-log Corp., Pennsylvania, U.S.A).

### Assessment of intracellular Ca^2+^ release

Washed human platelets (4×10^8^ cells/ml) were incubated with 3 μM fura-2-AM for 1 hour at 30°C. Unloaded fura-2-AM was removed by centrifugation (10 mins, 1000 *x*g) and platelets re-suspended in HEPES-Tyrodes buffer at 4×10^8^ cells/ml. To measure ADP-induced Ca^2+^ release, fura-2-AM was loaded into PRP. Changes in fluorescence were measured using a Tecan plate reader using excitation wavelengths of 340 nm and 380 nm.

### Western blotting

Samples were prepared and processed as described in [22, 23]

### Fluorescent labeling of free surface thiols

Using a method adapted from [24], washed platelets (100 μl at 4x10^8^/ml) were treated with compounds for 30 minutes at room temperature before the addition of DyLight-maleimide 488 (1 μM) for a further 15 minutes at room temperature, to label free thiols. Unbound dye was quenched with 2x reduced glutathione before samples were lysed by the addition of SDS-loading dye supplemented with 100 mM DTT and boiled for 10 minutes at 70°C.

### Thrombus formation under flow

The Bioflux200 system (Fluxion, South San Francisco, CA) was used to analyse thrombus formation in human whole blood under flow. Microchannels were coated with 0.1 mg·mL^−1^ collagen I (monomeric collagen from calf skin, Sigma, UK) for 1–2 hours at 37°C before blocking with 1% BSA in Tyrodes-HEPES buffer and washing with Tyrodes-HEPES buffer. Whole blood was incubated with drug or vehicle before the addition of 1 μM DiOC6 10 minutes before the blood was added to the wells. Thrombus formation was visualized by fluorescence microscopy at a shear rate of 1000 s^−1^. Representative pictures were taken at 10 min and surface area coverage was determined using Image J.

### Biotin labelling of free cell surface thiols and purification of biotinylated glycoproteins

Free cell surface thiols were alkylated with 2.5mM EZ-Link iodoacetyl-LC-biotin for 30 minutes at room temperature, after which free label was quenched with 5 mM reduced glutathione. Platelets were lysed with 0.5% SDS and centrifuged to remove cell debris. Biotinylated cell surface glycoproteins were isolated using a two-step affinity purification. Firstly glycoproteins were affinity purified from the lysates by binding to a 300 μl lentil lectin sepharose column, washing with 10 column volumes of PBS/0.01% Tx100 and eluting with 10% methyl-ɑ-D-gluco-pyranoside in PBS/0.01% Tx100. Secondly, biotin-labelled glycoproteins were isolated from the glycoprotein mixture by binding to a 300 ml monomeric avidin column, washing with 10 column volumes of PBS/0.01% Tx100 and elution with 5 mM biotin in PBS/0.01% Tx100. Samples were prepared from mass spectrometry as previously reported.

### Mass Spectrometry

After desalting on a C18 micro column, the samples were resuspended in 0.1% formic acid containing 2% acetonitrile and analysed on an Ultimate 3000 UHPLC (Dionex) coupled to a QExactive mass spectrometer (Thermo Fisher Scientific). Samples were injected directly on an in-house packed 25 cm C18 (Bishoff 3 micron bead diameter) column. Separation of peptides was achieved with the following gradient 5–30% buffer B over 90 min, 30–55% buffer B over 20 minutes and 98% buffer B for 5 minutes (buffer A: 0.1% formic acid, buffer B: 0.1% formic acid in acetonitrile) at a flow rate of 300nl/minute. Data were acquired in a data-dependent mode, automatically switching from MS to collision induced dissociation MS/MS on the top 20 most abundant ions with a precursor ion scan range of 350–1650 m/z. Full scan MS spectra were acquired at a resolution of 70,000 and MS/MS scans at 17,500 at a target value 3 x 106 and 1 x 105 ions respectively. Dynamic exclusion was enabled with exclusion duration of 40 seconds.

### Data analysis and quantitation

RAW data files were converted to the mzXML format and analysed using the to the University of Oxford in-house Central Proteomics Facilities Pipeline (CPFP version 2.1.2). The datasets from all three donors were combined but grouped within the software into DMSO control, PMX 464-treated and PX-12-treated. The data were searched with carbamidomethyl cysteine, oxidized methionine, deamidated asparagine/glutamine, and EZ-Link iodoacetyl-LC-Biotin as variable modifications. Precursor mass tolerance was set at ±20 ppm and MS/MS fragment ion tolerance at ±0.02 Da. Searches were performed against the Swissprot human database. All searches were performed against a concatenated target/decoy database, providing an empirical false discovery rate (FDR), results are reported at a 1 per cent target/decoy FDR for both peptides and proteins. SINQ at the protein level were performed on the grouped datasets to provide quantitative estimates of the relative protein abundance between DMSO treated and thiol inhibitor treated samples for proteins identified with at least two unique peptides.

### Statistical analysis of in vitro and ex vivo data

Statistical analysis was carried out using GraphPad Prism software. See Figure legends for specific details of analysis.

## Results

### Free thiols in platelet surface proteins are altered by Trx inhibitors

**Fig 1:**
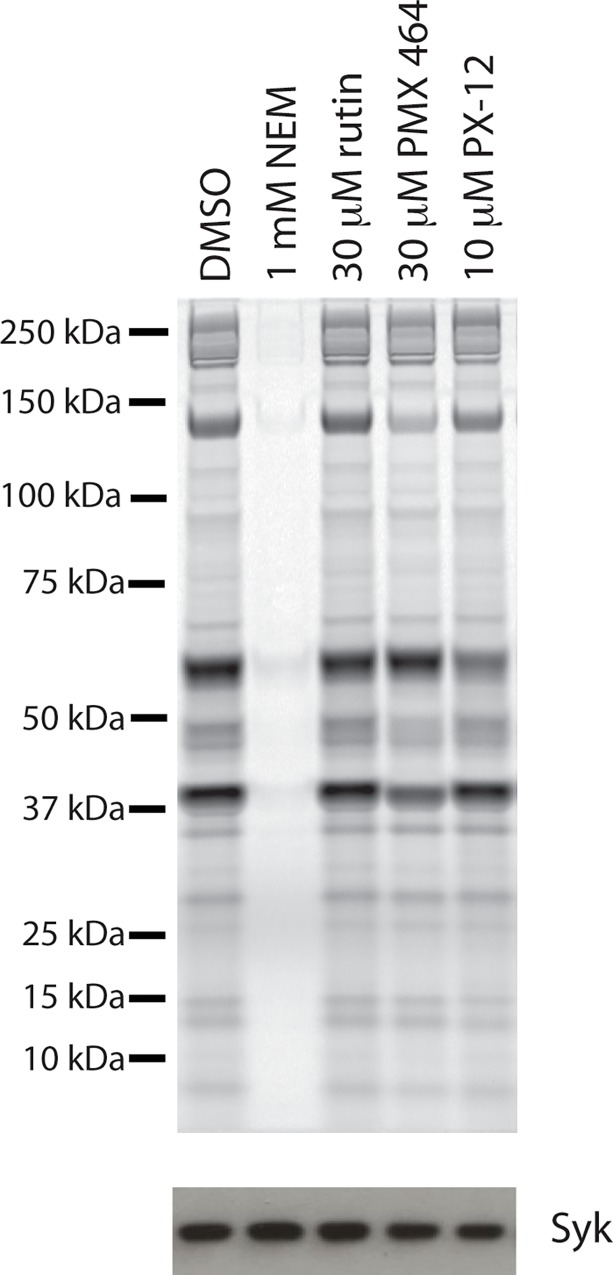
The free thiol profile at the surface of platelets is modified by inhibitors of Trx, but not PDI. Washed human platelets were incubated with compound (DMSO, rutin, PMX 464, PX-12 or NEM) for 30 minutes at 30°C before addition of 1 μM DyeLight-maleimide. Samples were quenched with 20 μM reduced glutathione before lysis and seperation by SDS-PAGE. DyeLight-maleimide 488 binding was visualised using a Typhoon imager. NEM (1mM), which reacts with free thiols, and was used as a negative control. Following scanning, samples were transfered onto nitrocellulose membrane and probed for Syk by Western blotting to assess gel loading.

Washed human platelets were incubated with Trx inhibitors (PMX 464 and PX-12), a PDI inhibitor (rutin), or vehicle alone (DMSO) for 30 minutes, before proteins containing free thiols were alkylated with the cysteine-reactive conjugate DyLight Maleimide-488, which is conjugated to the cell impermeant Alexa Fluor 488 dye. Free dye was quenched before samples were lysed and separated by SDS-PAGE; labeled proteins were visualized using a Typhoon Imager (Fig 1). N-ethylmaleimide (NEM) reacts with free thiols and was used as a control to demonstrate that we could detect a decrease in free thiols using this approach. Both PX-12 and PMX 464 (but not rutin) decreased the extent of free thiol labeling at the platelet surface. There were selective effects on some, but not all, protein species, suggesting that the two Trx inhibitors have different selectivity profiles.

### LC-MS/MS reveals a number of potential thioredoxin substrates at the cell surface of resting platelets

We next employed an unbiased mass spectrometry approach to identify proteins whose redox profile was affected by PX-12 and PMX 464. Washed human platelets from three donors were incubated with Trx inhibitors or vehicle (DMSO) before labeling with the sulfhydryl-reactive compound iodoacetyl-LC-biotin. The abundance of biotin-labeled peptides in the treatment groups was compared to that of the control group (DMSO) and is represented as a ratio (Table 1). A value <1 indicates that drug treatment reduces the abundance of a given free thiol-containing peptide (red), while a value >1 indicates that abundance increases (green). Generally, PMX 464 decreases free thiols (ratio <1) more consistently than PX-12, but both inhibitors reduce the abundance of free thiols overall. Interestingly, GPVI peptides were identified, as were a number of peptides from integrins, as well as vWF and its receptor complex.

**Table 1:** Trx inhibitors alter the abundance of peptides with free thiols on the surface of resting human platelets.

						SINQ ratios	
Swissprot ID	Gene	Protein Description	PSMs	Unique Sequences	% Coverage	PMX 464	PX-12
**Immunoglobulin superfamily domain containing proteins**							
A0A075B738	PECAM1	Platelet endothelial cell adhesion molecule	112	26	44.3	0.15	0.36
Q96AP7	ESAM	Endothelial cell-selective adhesion molecule	55	11	36.7	0.05	0.55
O95866-2	G6B	Isoform A of Protein G6b	38	6	30.5	0.07	0.74
Q15762	CD226	CD226 antigen	32	8	40.5	0.23	0.61
Q9BX67	JAM3	Junctional adhesion molecule C	24	4	12.9	0.15	0.24
Q5VY43	PEAR1	Platelet endothelial aggregation receptor 1	20	6	6.4	2.69	9.71
Q86YW5	TREML1	Trem-like transcript 1 protein	15	4	19	0.31	0
P19320-2	VCAM1	Vascular cell adhesion protein 1	13	7	11.1	0.07	1.22
Q9HCN6-2	GP6	Platelet glycoprotein VI	4	2	23.1	0	0.11
**Selectins**							
P16109	SELP	P-selectin	948	33	45.6	0.47	3.4
P14151-2	SELL	Isoform 2 of L-selectin	13	3	4.8	0.86	6.04
					
**Cell adhesion molecules**							
Q9Y624	F11R	Junctional adhesion molecule A	77	12	43.5	0.14	0.72
P18433-2	PTPRA	Receptor-type tyrosine-protein phosphatase alpha	20	9	13.6	0.01	0.57
P16284-2	PECAM1	Isoform 12 of Platelet endothelial cell adhesion molecule	18	26	45.6	0.23	0.85
O43866	CD5L	CD5 antigen-like	4	2	5.2	0	1.91
**Von Willibrand factor interacting proteins**							
P04275	VWF	von Willebrand factor	1034	86	35.2	0.31	1.08
P07359	GP1BA	Platelet glycoprotein Ib alpha chain	761	22	28.1	0.2	0.89
P40197	GP5	Platelet glycoprotein V	384	21	55	0.15	0.67
P14770	GP9	Platelet glycoprotein IX	63	5	30.5	0.24	1.04
**Semaphorins and plexins**							
Q9ULL4-2	PLXNB3	Isoform 2 of Plexin-B3	243	38	26.5	0.21	1.13
O15031	PLXNB2	Plexin-B2	160	27	19.6	0.35	0.9
Q92854	SEMA4D	Semaphorin-4D	148	17	23	0.16	0.5
Q9HCM2	PLXNA4	Plexin-A4	128	35	22	0.18	0.99
P98172	EFNB1	Ephrin-B1	25	7	26.6	0.04	0.58
Q6UX71	PLXDC2	Plexin domain-containing protein 2	20	5	11	0.04	1.89
Q13591	SEMA5A	Semaphorin-5A	14	5	4.7	0.07	0.28
**Integrins**							
P08514	ITGA2B	Integrin alpha-IIb	1267	40	48.2	0.22	0.59
P05556	ITGB1	Integrin beta-1	780	43	52.8	0.28	1.2
P23229-3	ITGA6	Isoform Alpha-6X1B of Integrin alpha-6	407	45	54.7	0.18	0.68
P05106	ITGB3	Integrin beta-3	352	32	43.9	0.1	0.59
P17301	ITGA2	Integrin alpha-2	220	37	44.2	0.09	0.45
P18084	ITGB5	Integrin beta-5	156	19	24.5	0.28	0.76
P08648	ITGA5	Integrin alpha-5	37	11	13	0.04	0.17
**Redox related**							
P07237	P4HB	Protein disulfide-isomerase	232	28	52.2	0.23	0.36
P30041	PRDX6	Peroxiredoxin-6	230	17	79.5	0.42	1.46
P30101	PDIA3	Protein disulfide-isomerase A3	198	26	55.4	0.13	0.58
Q06830	PRDX1	Peroxiredoxin-1	118	10	49.2	0.68	0.78
P32119	PRDX2	Peroxiredoxin-2	88	11	42.4	0.62	0.87
Q15084-2	PDIA6	Isoform 2 of Protein disulfide-isomerase A6	81	11	27.9	0.08	0.36
Q8NBM8	PCYOX1L	Prenylcysteine oxidase-like	66	5	11.1	0.24	1.09
P10599	TXN	Thioredoxin	50	6	51.4	1.19	1.11
Q13162	PRDX4	Peroxiredoxin-4	48	6	36	0.79	2.14
P13667	PDIA4	Protein disulfide-isomerase A4	46	16	27.9	0.17	0.43
O43396	TXNL1	Thioredoxin-like protein 1	20	6	27	0.05	0.4
Q86YB8	ERO1LB	ERO1-like protein beta	18	8	20.1	0.03	0.17
P49908	SEPP1	Selenoprotein P	12	3	8.4	0.1	0.84
Q9H3N1	TMX1	Thioredoxin-related transmembrane protein 1	10	3	11.1	0.09	0
Q8NBS9-2	TXNDC5	Isoform 2 of Thioredoxin domain-containing protein 5	9	4	14.5	0	2.04
P35754	GLRX	Glutaredoxin-1	6	2	11.3	0.43	0.43
Q96JJ7	TMX3	Protein disulfide-isomerase TMX3	4	4	8.6	0	0
**Other**							
P07996	THBS1	Thrombospondin-1	1379	62	62.7	0.14	0.62
Q02413	DSG1	Desmoglein-1	521	35	45	1.19	0.68
O14672	ADAM10	ADAM 10	57	8	11.9	0.16	1.22
P40238	MPL	Thrombopoietin receptor	48	8	13.1	0.1	0.84
P01033	TIMP1	Metalloproteinase inhibitor 1	38	4	22.7	0.16	0.58
P02775	PPBP	Platelet basic protein	29	5	27.3	0.43	2.85

### Inhibition of Trx, but not PDI, had concentration-dependent effects on agonist-induced intracellular Ca^2+^ release

**Fig 2:**
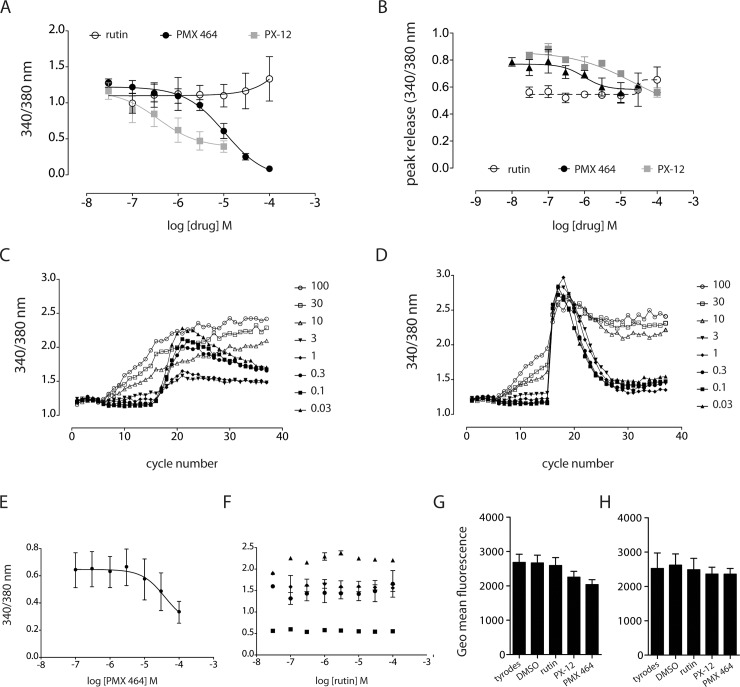
GPVI-mediated Ca^2+^ release is sensitive to inhibitors of Trx-R and Trx. Washed human platelets were loaded with 3 μM fura2-AM and Ca^2+^ release monitored at 340nm and 380nm. Fluorescence was monitored for 5 cycles to obtain a baseline reading, following which drug was added for approx. three minutes for a further 10 cycles, before addition of agonist (1 μg/ml CRP, 0.5 U/ml thrombin, 0.3 ∞M U46619, or 20 μM ADP). PMX 464 and PX-12 inhibited CRP-XL-induced Ca2+ release in a concentration-dependent manner (A), with a modest, but non-significant effect on Ca2+ release induced by ADP (B). PX-12 induced Ca2+ release independently of agonist at or above concentrations of 10 μM (C and D). PMX 464 inhibited Ca2+ release by U46619 at 0.1μM (E, R^2^ 0.6), but not at 0.3 μM or 1 μM. Rutin had no effect on Ca2+ release by any agonist (F, thrombin, black triangles; CRP-XL black circles; U46619, black diamonds; ADP, black squares). To determine whether Trx and PDI inhibitors were inducing receptor shedding, whole blood was incubated with drug (30 μM rutin, 10 μM PX-12, 30 μM PMX 464) or vehicle for 10 minutes (G) or 30 minutes (H), to which was added anti-GPVI antibody conjugated to AlexaFluor 647 to quantify cell surface levels for GPVI by flow cytometry (n = 4, +SEM, one-way ANOVA, Bonferroni post-hoc test).

Having found that Trx inhibitors altered the prevalence of thiol-labeled peptides, including those relating to proteins involved in platelet adhesion and activation, we sought to experimentally verify these findings by assessing the effects of these compounds on platelet function. In the first instance, inhibitors of PDI (rutin) or Trx (PX-12 and PMX 464) were added to fura2-loaded washed human platelets (3 mins) prior to the addition of agonist to determine whether Trx inhibitors affected changes in the release of intracellular Ca^2+^ upon activation. PMX 464 demonstrated a clear concentration-dependent inhibition of intracellular Ca^2+^ release in response to the GPVI-specific agonist, CRP-XL, (IC_50_ ~10 μM, R^2^ 0.93, Fig 2A), with PX-12 also inhibited CRP-XL-induced Ca^2+^ release in a concentration-dependent manner (IC_50_ ~1 μM, R^2^ 0.5, Fig 2A). Both Trx inhibitors, at high concentrations (100 μM), reduced cytosolic Ca^2+^ release in response to ADP by ~20% from maximum, but this was not a statistically significant change (Fig 2B). As shown in Fig 2C and 2D, PX-12 at concentrations ≥10 μM induced agonist-independent increases in intracellular Ca^2+^ release. It is noteworthy that PMX 464 had some minor effect on U46619-induced Ca^2+^ release, but these effects were only evident at low U46619 concentrations (0.1 μM), Fig 2E, IC_50_ 20 μM, R2 = 0.6). Rutin had no effect on Ca^2+^ release by any agonist (Fig 2F). The data presented here suggest that the GPVI-ligand interaction, or some aspect of GPVI function, is redox labile and positively regulated by Trx, as loss of Trx activity reduces CPR-XL-induced intracellular Ca^2+^ release. To ensure that these effects were not attributable to a of loss of GPVI from the cell surface, fluorescently-labeled HY101 anti-GPVI monoclonal antibody was used to quantify surface levels of the receptor. Whole blood samples were incubated with drug (30 μM rutin and PMX 464, or 10 μM PX-12) or vehicle for either 10 (Fig 2G) or 30 minutes (Fig 2H), then fixed and assessed for anti-GPVI antibody binding to GPVI at the platelet surface by flow cytometry. Surface levels of GPVI were not altered by vehicle or drug at either time point.

### Kinetics is a determining factor for PMX 464-mediated inhibition of CRP-XL-induced aggregation in platelet-rich plasma

**Fig 3:**
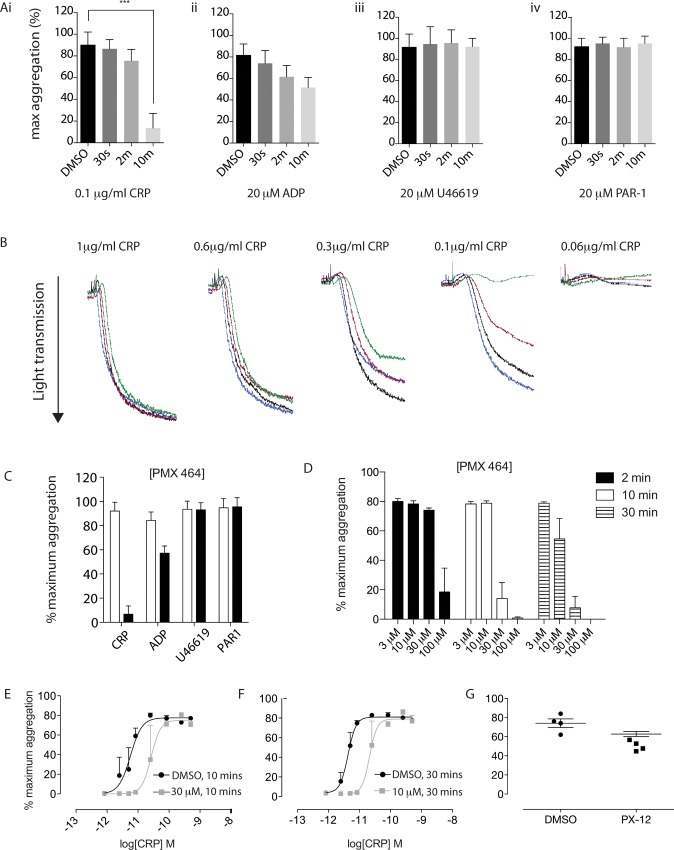
PMX 464 selectively inhibits CRP-XL-induced aggregation in PRP in a time-dependent manner. PRP isolated from healthy human volunteers was incubated with 30 μM PMX 464 or vehicle (DMSO) for 30s, 2m or 10m at room temperature before being assess by light transmission aggregometry (A); example traces are shown in B. PMX 464 selectively and significantly inhibited CRP-XL-induced aggregation with a 10 minutes incubation (C, DMSO in white, PMX 464 in black, n = 4, +SEM, 2-way ANOVA). Increasing the incubation time of PMX 464 potentiated the level of inhibition seen with CRP-XL-induced PRP aggregation (D, black bars = 2 minutes, white bars = 10 minutes, horizontal stripes = 30 minutes). As shown in E and F, a 10 min incubation with 30 μM PMX 464 increased the EC50 of CRP-XL ~5-fold (E, 8 pM to 40 pM, p = 0.0316, two-tailed t-test with Bonferroni correction), while a 30 minute incubation with 10 μM PMX 464 increased the EC_50_ of CRP-XL by 5pM to 30pM (~6-fold, p = 0.0334, two-tailed t-test with Bonferroni correction). PX-12 (30 minutes, 3 μM PX-12) did not inhibit CRP-XL-induced PRP aggregation (G; n = 4, p = 0.1, 2-tailed t-test with Bonferroni correction).

To determine whether the effects on CRP-XL-induced Ca^2+^ release translated to an impairment of physical aggregation, platelet-rich plasma (PRP) was isolated from whole blood and incubated with drug before being assessed by light transmission aggregometry. CRP-XL-induced aggregation (0.1 μg/ml) was severely and significantly impaired (>90% inhibition, n = 4, p<0.0001) following a 10 min incubation with 30 μM PMX 464 (Fig 3Ai); shorter incubations also reduced aggregation, but were less dramatic and not significant. There was also a small but non-significant effect on aggregation induced by ADP at 30 minutes (Fig 3Aii), but no effect on PAR1- or U46619-induced aggregation (Fig 3Aiii and 3Aiv, respectively). Example aggregation traces for CRP-XL are shown in Fig 3B (10 minute incubation) with selectivity and time-dependent effects summarised in Fig 3C and 3D. Further evidence that the effects of PMX 464 comprise a temporal component, Fig 3E and 3F shows that a 10 minute incubation with 30 μM PMX 464 increases the EC_50_ of CRP-XL to a similar extent as a 30 minute incubation with 10 μM PMX 464, suggesting that lower doses of drug can reach the same level of inhibition as higher doses if the incubation period is extended. Inhibition of CRP-XL-induced aggregation by PMX 464 was attenuated by the addition of an equimolar concentration of recombinant Trx (S1 Fig). PX-12 was unable to induce the same degree of inhibition, even at 30-minutes (Fig 3G). Production of reactive oxygen species (ROS), which is released following GPVI activation, was also reduced by PMX 464 in a concentration-dependent manner (S2 Fig).

### Inhibition of Trx inhibits thrombus formation in whole blood under flow, ristocetin-induced platelet agglutination, and clot retraction

**Fig 4:**
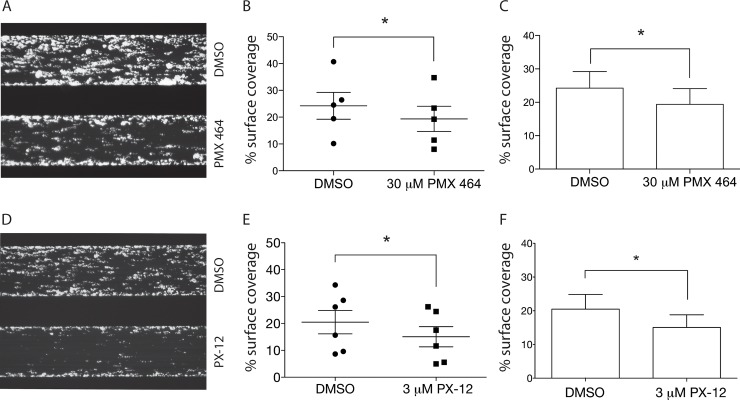
PMX 464 and PX-12 inhibit thrombus formation over Type I collagen in whole blood under flow conditions. Images of the channels are shown in A (30 μM PMX 464) and D (3 μM PX-12) and quantified to show variation between donors for both drugs (B and E, respectively), as well as an overall summary (C and F, respectively), n = 5, +/- SEM, 2-tailed t-test with Bonferroni correction.

Based on our *in vitro* observations that PMX 464 inhibited CRP-XL-induced Ca^2+^ release and aggregation, we hypothesized that PMX 464, and possibly PX-12, would attenuate thrombus formation on collagen in whole blood under flow. Whole blood was incubated with drug for 30 minutes at room temperature prior to being subjected to flow conditions approximating arterial shear (1000s^-1^). Both PX-12 (3 μM, Fig 4A) and PMX 464 (30 μM, Fig 4D) reduced thrombus formation on type I collagen under flow conditions. Quantification of thrombi containing DiOC6-labelled platelets revealed a ~30% reduction in surface coverage for both inhibitors compared to DMSO control (PX-12 p = 0.002, Fig 4B and 4C; PMX 464 p = 0.024, Fig 4E and 4F).

**Fig 5:**
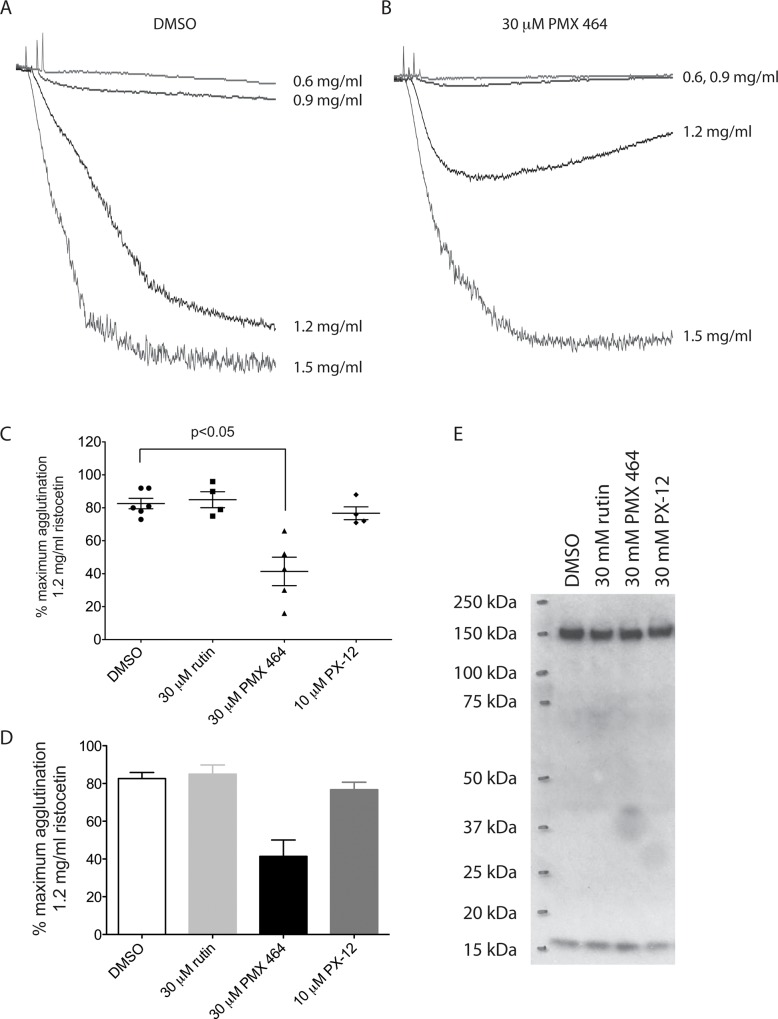
PMX 464 attenuates ristocetin-induced platelet agglutination. PMX 464 (30 μM) reduced platelet agglutination induced by ristocetin in platelet rich plasma (A-D, n = 5, +/- SEM, one-way ANOVA, Bonferroni multiple comparisons test). Samples of washed platelets incubated with drug or vehicle were separated by SDS-PAGE and levels of GPIb assessed by Western blotting (E).

As shown in Table 1, PMX 464 reduced the abundance of free thiol-containing peptides isolated from vWF and its receptor complex. To determine whether this translated into direct effects on the vWF-GPIb interaction, ristocetin-induced platelet agglutination was carried out. As can be seen from Fig 5, pre-treating platelet-rich plasma with PMX 464 reduced platelet agglutination induced by ristocetin (B) compared to DMSO alone (A). PX-12 and rutin had no effect (Fig 5C and 5D), consistent with the data in Table 1 and in Fig 3. To ensure that loss of ristocetin-induced vWF-GPIb binding observed following PMX 464 incubation is not due to the loss of GPIb from the cell surface, samples from Fig 1 were again separated by SDS-PAGE as assessed for GPIb levels. As can be seen in Fig 5E, the high and low molecular weight species corresponding to GPIb were unaltered by incubation with inhibitors when compared to DMSO (n = 3). Peptides corresponding to the ɑIIbβ3 integrin were also identified in the proteomics experiment and we observed an inhibition of clot retraction in PRP by PMX 464 (S3 Fig).

## Discussion

Post-translational modification of proteins allows for rapid changes in protein activity without lengthy and energetically expensive *de novo* synthesis. It underscores a plethora of essential cellular processes and allows for incredibly rapid responses to spatiotemporal cues. There are a considerable number of different types of post-translational modifications including phosphorylation, methylation, nitrosylation, sulfation, and reduction/oxidation of disulphide bonds, among others. Regulation of disulphide bond chemistry is not only essential for the proper folding of proteins in the endoplasmic reticulum, but can also regulate protein activity and function[25]. Allosteric disulphide bonds contribute to the regulation and fine tuning of essential biological processes, including thrombosis and inflammation, and appropriate reduction/oxidation biochemistry is key to maintaining cell function and viability, and changes in this balance underscores myriad pathophysiological conditions[14, 15].

There is increasing evidence that thiol isomerases play significant roles in thrombosis and haemostasis, mainly through effects on plasma proteins[13, 26] and integrins[14, 27], and to date, PDIs have been the most intensively studied in this regard [12, 27–30]. Trx is another thiol isomerase that can reduce a broad range of substrates, from small molecules to proteins. Together with peroxiredoxin, Trx-1 can scavenge hydrogen peroxide as well as directly reducing protein disulphide bonds to affect protein structure and/or activity (e.g. HIF1a[31], p53[32], CD4[7, 8], CD132[11], PDI[33], MPK38[17], ASK1[34], and the C-propeptide region of human pro ɑ1 type I collagen[35]). Known to be important in cancer pathology, as well as inflammation, cytokine signalling [11, 36], and HIV infection via gp120[7], its role in thrombosis and haemostasis remains largely unknown. Levels of Trx have been reported in platelets[37] and exceed that reported for PDI or the closely related ERp proteins, and we set out to test the hypothesis that Trx, like PDI, affects platelet function. We found that inhibition of Trx with small molecule inhibitors decreases the abundance of proteins containing free thiols on the surface of resting platelets. Then, using a proteomics approach, we found that, more specifically, treatment of platelets with PMX 464 and PX-12 decreased the abundance of free thiol-containing peptides from a number of surface proteins involved in adhesion and activation, including GPVI. This data was then validated using a series of platelet functional assays that demonstrated that these drugs, particularly PMX 464, inhibited GPVI-mediated platelet activation and thrombus formation, as well as the interaction of vWF with its receptor complex, as evidenced by an impairment of risctocetin-induced vWF-GPIb binding. Our data suggests that thiol reductases, particularly Trx, maintain a basal level of free thiols on the resting platelet surface that is essential for activation. It should be noted that PX-12 and PMX 464 are commercially available Trx inhibitors, but their selectivity has not been clearly defined; the differences we see between these drugs in the proteomics and functional data could be attributable to differences in their selectivity profiles (i.e. for other oxidoreductases), or related to their potency for Trx itself. To undertake a fully comprehensive study of these drugs in terms of selectivity and efficacy is beyond the scope of this study, but it is important to bare this in mind when considering the data. Regardless, it was previously thought that secretion of thiol reductases/isomerases post-activation was the key redox event in thrombus formation, but our data, for the first time, highlights a novel role for redox regulation *pre*-activation, as Trx inhibitors disable platelet responses to key activating factors. This is in contrast to other biological systems, such as the immune system, where reduction of allosteric disulphides such as CD132 and CD44 inhibits receptor function[11, 38].

This study not only highlights the importance of allosteric disulphides to the reactivity of resting platelets, but the functional data shown above also validates the use of global proteomics approaches to identify proteins that are redox labile.

Trx inhibitors showed selectivity for the adhesion/activation receptors GPVI and GPIb; we saw minor effects on responses to ADP at high concentrations of drug, as well as an inhibition of Ca^2+^ release induced by low concentrations of the thromboxane A2 receptor (TPR) agonist U46619, but these were not significant. Both TPR and P_2_Y_12_ both have labile disulphide bonds that could be affected by reduction/oxidation at the cell surface. Indeed, TPR has been shown to be redox labile[39], while P_2_Y_12_ has a labile disulphide (Cyc97-Cys175) known to undergo covalent modification by the active metabolites of P_2_Y_12_ antagonists[40].

We also observed a temporal effect for the efficacy of PMX 464. Longer incubations enhanced the inhibitory effect of this drug in a GPVI-selective manner. PMX 464 is a Trx inhibitor, and not, to the best of our knowledge, a GPVI antagonist, which may account for the time-dependency we observe; substrates of the NADPH/Trx-R/Trx system will be reduced when the system is active but will not immediately be inactivated when the system is impaired.

The impairment of GPVI and GPIb activity is not due to loss of these receptors from the cell surface as levels of GPVI are unchanged by drug incubation, and GPIb showed no evidence of shedding. The fact that inhibition is observed in washed platelets as well as PRP and whole blood implies that loss of receptor function is also not due to the alkylation of free thiols by plasma components, as alkylating agents present in blood plasma, such as homocysteine and glutathione, will have been removed.

In addition to changes in the abundance of thiol-containing peptides from GPVI and GPIb, changes in other proteins known to be important for platelet activity and function were observed (ADAM10, TPO receptor, G6b, TREM-like transcript-1, PECAM-1, thioredoxin domain-containing proteins including PDI and ERp57), as well as those involved in adhesion and activation (GPV, GPIX, the ɑ2b and β3 integrins, JAMs, etc). This indicates that maintenance of basal thiol levels in a number of platelet surface proteins may influence reactivity and function, but their contribution to overall platelet activity will require further investigation.

Redox regulation of allosteric disulphide bonds in the proteins listed in Table 1 indicate that Trx modulates platelet reactivity and supports thrombus formation, especially with regards to GPVI and GPIb. The fact that PMX 464 and PX-12 have little or no effect on platelet responses to thrombin, ADP, or U46619 suggests that Trx activity is not required for the later stages of thrombus formation (growth and stability), for which PDI is known to be functionally important. Selective targeting these oxidoreductases may offer the opportunity to temporally modulate thrombus formation.

This study demonstrates a clear and profound effect on GPVI-induced platelet activation and aggregation, both in washed platelets, PRP, and a whole blood setting by Trx inhibitors, as well as effects on GPIb. This indicates that the NADPH/thioredoxin reductase/thioredoxin system helps to ensure that platelets are reactive to collagen, supporting adherence and activation at sites of injury and the initiation of thrombus formation. This selective effect could be of clinical benefit if inhibiting the GPVI-collagen or vWF-GPIb interaction with small molecules lives up to the promise shown by biological agents, especially with regards to ischemic stroke[41–44].

## Supporting Information

S1 Fig Recombinant reduced Trx attenuates PMX 464-mediated inhibition of CRP-XL- induced aggregation in PRP. PMX 464 inhibits CRP-XL-induced aggregation of PRP, with a concomitant increase in the EC50 of CRP-XL and a rightward shift of the curve (A, n = 6, +/- SEM). Addtion of equimolar recombinant reduced Trx just prior to drug addition attenuates the effects of PMX 464 (A and B, n = 4–6, one-way ANOVA, Barlett’s test for equal variances post hoc test).
(EPS)

S2 Fig PMX 464 attenuates reactive oxygen species generation induced by CRP-XL. Reactive oxygen species (ROS) was detected using the ROS dye H2DCFDA, and production of fluorescent DCF was detected using a plate reader assay format. CRP-XL induces ROS generation in a dose dependent manner, and this is attenuated by PMX 464 (A, n = 3, +SEM). PMX 464 significantly reduces ROS formation at high (10 μg/ml CRP-XL, B) and moderate (1 ∞g/ml CRP-XL, C). Sample size (n) = 3, +SEM, 1-way ANOVA, Bonferroni multiple comparison post hoc test (* p < 0.05, ** p < 0.01).
(EPS)

S3 Fig PMX 464 attenuates clot retraction. PMX 464 inhbited clot retraction in PRP (n = 4, +/- SEM, one-way ANOVA, Bonferroni correction).
(EPS)

## Abbreviations

Trx: thioredoxin
Trx-R: thioredoxin reductase
PDI: protein disulphide isomerase
GPVI: glycoprotein VI
GPIb: glycoprotein Ib
vWF: von Willebrand factor
DMSO: dimethylsulfoxide
CRP-XL: collagen-related peptide, cross-linked)
PRP: platelet-rich plasma
PAR: protease-activated receptor
